# Reciprocal ECG change in ST-elevation myocardial infarction is associated with area at risk and myocardial salvage following revascularization

**DOI:** 10.1186/1532-429X-15-S1-P172

**Published:** 2013-01-30

**Authors:** Ananth Kidambi, Adam N Mather, Akhlaque Uddin, Manish Motwani, David P Ripley, Bernhard A Herzog, Julian Gunn, Sven Plein, John P Greenwood

**Affiliations:** 1 Department of Cardiology, Multidisciplinary Cardiovascular Research Centre & Leeds Institute of Genetics, Health and Therapeutics, Leeds, UK; 2 Hull and East Yorkshire Cardiothoracic Centre, Castle Hill Hospital, Kingston-upon-Hull, UK; 3 Department of Cardiovascular Science, University of Sheffield, Sheffield, UK; 4 Department of Cardiology, Sheffield Teaching Hospitals NHS Foundation Trust, Sheffield, UK

## Background

ST elevation acute myocardial infarction (STEMI) is frequently associated with reciprocal ST depression in the contralateral ECG leads. The ECG remains a primary diagnostic factor in the hyperacute treatment of AMI. There has been longstanding debate over the relevance of reciprocal ECG changes in AMI.

CMR can retrospectively determine myocardial area at risk (AAR), namely, the area of myocardium that is susceptible to infarction prior to opening the infarct-related artery. T2-weighted (T2w) imaging can be combined with measures of infarction by late gadolinium enhanced (LGE) imaging to derive myocardial salvage, a measure with established prognostic relevance. We hypothesised that reciprocal ECG change reflects larger AAR as defined by CMR.

## Methods

Patients after primary percutaneous coronary intervention for first STEMI underwent CMR at 1.5T within 3 days of reperfusion. Presenting ECGs were assessed for presence or absence of reciprocal change, defined as ≥1mm ST depression in ≥2 inferior leads for anterior STEMI, or ≥2 anterior leads for inferior STEMI. Patients with posterior ECG changes were excluded from analysis. Infarcted tissue was defined as hyperenhancement ≥2 standard deviations (SD) above remote myocardium on LGE imaging. AAR was defined on T2w images, defined as the mass of myocardium with signal intensity ≥2 SD above remote, normalized to LV myocardial mass. Myocardial salvage was calculated as (AAR mass - infarct mass), and myocardial salvage index as (myocardial salvage / AAR mass).

**Figure 1:**
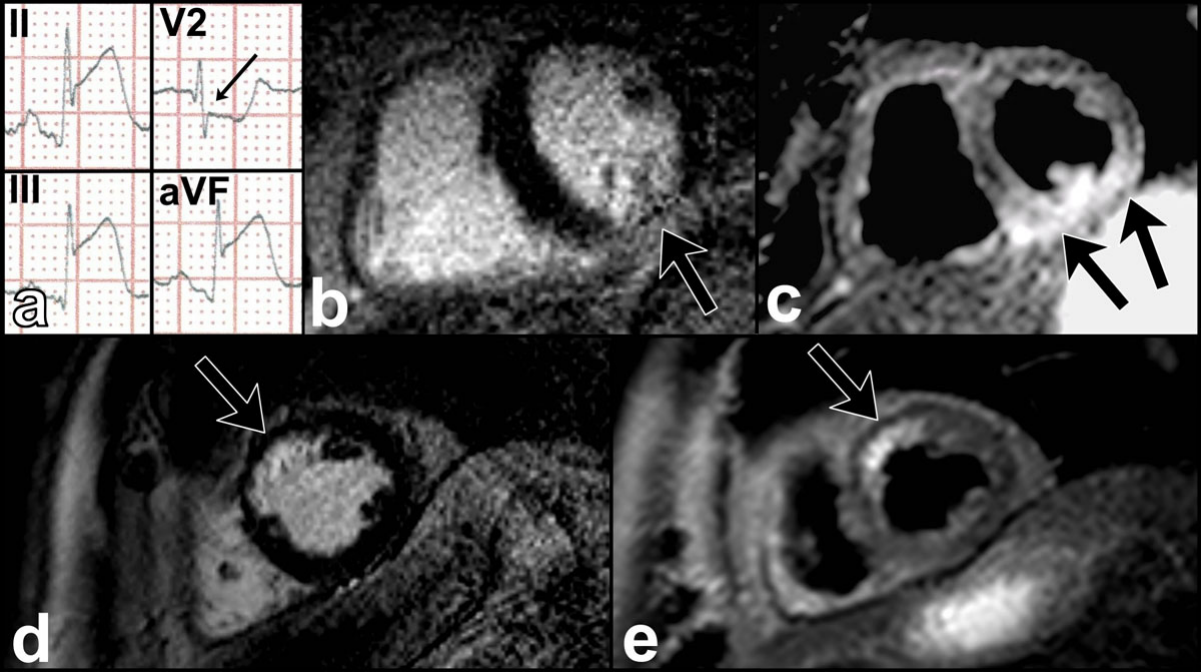
Comparison of AAR and infarct size in patients with and without reciprocal ECG changes. Sample ECG of inferior AMI with reciprocal change (a, arrowed) corresponds to an infarct zone on LGE imaging (b) with relatively large AAR on T2w imaging (c). A different patient without reciprocal ECG change has infarct (d) and AAR (e) of similar size.

## Results

Thirty-five patients had ECGs and images suitable for analysis. There were no significant differences in patient demographics (Table 1). Patients with reciprocal ECG changes had significantly higher AAR than those without (42.3% vs. 23.6%, p<0.001), myocardial salvage (17g vs 2g, p<0.001) and myocardial salvage index (40% vs. 7%, p<0.001). Infarct size was not significantly different between patients with and without reciprocal ECG changes (25.6g vs. 27.4g, p=0.8), nor was ejection fraction (43% vs. 45%, p=0.5) or LV end diastolic volume (173ml vs. 174ml, p=0.9).

**Table 1:** Patient demographics.

	No reciprocal ECG change (n=16)	Reciprocal ECG change (n=19)
**Age (years)**	58 ± 3	57 ± 2
**Male sex**	15 (94%)	16 (84%)
**Diabetes**	0 (0%)	3 (16%)
**Cholesterol (mmol/l)**	5.9 ± 0.2	5.6 ± 0.5
**Pain onset to <br/> revascularization time (min)**	237 ± 35	230 ± 29
**TIMI 3 at end of procedure**	15 (94%)	17 (89%)
**Body surface area**	2.0 ± 0.03	2.0 ± 0.04

Data are shown as mean ± SEM or n (%). There were no significant differences between the groups (p≥0.1 for all).

## Conclusions

Patients with STEMI presenting with reciprocal ECG changes have significantly larger AAR, and significantly higher myocardial salvage and salvage index than those without. Reciprocal ECG changes may be a marker of larger volumes of myocardium at risk, and may also denote patients with larger potential for salvage with revascularization. There was no relationship to infarct size, presumably as other factors, such as time to revascularization and TIMI flow post-procedure will influence the degree of myocardium infarcted.

## Funding

S.P is funded by British Heart Foundation fellowship (FS/10/62/28409)

S.P and J.P.G receive a research grant from Philips Healthcare

